# Iron Absorption from Two Milk Formulas Fortified with Iron Sulfate Stabilized with Maltodextrin and Citric Acid

**DOI:** 10.3390/nu7115448

**Published:** 2015-10-30

**Authors:** Fernando Pizarro, Manuel Olivares, Eugenia Maciero, Gustavo Krasnoff, Nicolas Cócaro, Diego Gaitan

**Affiliations:** 1 Micronutrients Laboratory, Institute of Nutrition and Food Technology (INTA), University of Chile, Avda, El Líbano 5524, 6903625 Santiago, Chile; molivare@inta.uchile.cl; 2 Kasdorf S.A. Av. Panamericana y Gral. Savio (1619) Garín, Buenos Aires, Argentina; eugemaciero@hotmail.com (E.M.); gustavo.krasnoff@danone.com (G.K.); nicolas.cocaro@danone.com (N.C.); 3 Escuela de Nutrición y Dietética-Universidad de Antioquia, UdeA, Calle 70 No. 52-21, Medellín, Colombia; diego.gaitan@udea.edu.co

**Keywords:** iron bioavailability, milk formula, toddlers, iron fortification

## Abstract

Background: Fortification of milk formulas with iron is a strategy widely used, but the absorption of non-heme iron is low. The purpose of this study was to measure the bioavailability of two iron fortified milk formulas designed to cover toddlers’ nutritional needs. These milks were fortified with iron sulfate stabilized with maltodextrin and citric acid. Methods: 15 women (33–47 years old) participated in study. They received on different days, after an overnight fast, 200 mL of Formula A; 200 mL of Formula B; 30 mL of a solution of iron and ascorbic acid as reference dose and 200 mL of full fat cow’s milk fortified with iron as ferrous sulfate. Milk formulas and reference dose were labeled with radioisotopes ^59^Fe or ^55^Fe, and the absorption of iron measured by erythrocyte incorporation of radioactive Fe. Results: The geometric mean iron absorption corrected to 40% of the reference dose was 20.6% for Formula A and 20.7% for Formula B, versus 7.5% of iron fortified cow’s milk (*p* < 0.001). The *post hoc* Sheffé indeed differences between the milk formulas and the cow’s milk (*p* < 0.001). Conclusion: Formulas A and B contain highly bioavailable iron, which contributes to covering toddlers’ requirements of this micronutrient.

## 1. Introduction

Iron deficiency is one of the most prevalent nutritional deficiencies worldwide. Infants, pre-school and school children, as well as women of child-bearing age and pregnant women are the most vulnerable groups [1].

Experts agree that food fortification is the best long-term strategy to prevent iron deficiency in the population in general [2]. However, there is a variety of relevant technical considerations regarding the use of iron as a fortifier. In the first place, the food vehicle must be consumed on a regular basis by the target population. In the second place, the absorption of fortifying iron must be regulated by the iron nutritional status of the subject; otherwise, there may be a potential risk of overload; this regulation has been described for both heme and non-heme iron [3]. In the third place, the fortificant must be bioavailable.

From the nutrition standpoint, iron bioavailability has been shown to be more important than the iron content of a certain food. Over 95% of dietary iron is found as non-heme iron, the absorption of which is influenced by enhancers such as ascorbic acid [4] or inhibitors such as tannins [5], phytates [6], and calcium [7].

The main food source during early life is milk. However, this food has very low quantities of iron. Cow’s milk has between 0.40 and 0.59 mg of iron/L and human milk has between 0.20 and 0.69 mg of iron/L [8]. Despite this similarity, 49% of the iron bioavailability of human milk is significantly higher than 19% of cow’s milk [9]. This lower bioavailability of iron in cow’s milk has mainly been attributed to calcium and to the proteins contained in this food [10]. When cow’s milk is fortified with 15 mg of iron as ferrous sulfate, its bioavailability is reduced to 4%–6% and it is doubled when ascorbic acid is added in a molar ratio to iron of 2:1 [11].

Due to ethical constraints, and due to the use of radioisotopes, we use women as a surrogate for infants. Hurrell *et al.*, have demonstrated that results obtained in adults on Fe absorption from infant formulas can be extrapolated to infants [12].

The purpose of this study was to measure the bioavailability of two iron fortified milk formulas designed to cover the nutritional needs of toddlers. These milks were fortified with iron sulfate stabilized with maltodextrin and citric acid.

## 2. Experimental Section

**Subjects:** Apparently healthy females between 35 and 46 years old using a birth control method (e.g., intrauterine device, oral contraceptive, or tube ligation) were recruited by local advertisements. The subjects were informed of study details and the first 15 women that agreed to participate in the study were selected. All of them signed a written informed consent previously approved by the Ethical Committee of the Institute of Nutrition and Food Technology (INTA) of the University of Chile (Approval Act No. 35, Wednesday, 13 November 2013). The Chilean Commission on Nuclear Energy approved the doses of radioisotopes used. None of the women were pregnant, as confirmed by a negative test for human chorionic gonadotropin in urine. None had consumed any vitamin or mineral supplement in the previous six months.

**Study design:** Formula contents of total protein, casein, maltodextrin, calcium, iron, ascorbic acid, and prebiotic are given in Table 1. Milk based formulas and cow’s milk in powdered form were prepared according to manufacturer instructions and tested. Formula A (Nutrilon 3^®^) and Formula B (Vital 3^®^) were manufactured by Kasdorf S.A, Buenos Aires, Argentina. Non-fortified Cow’s milk was prepared by Milkaut S.A., Santa Fe, Argentina.

**Table 1:** Chemical composition of the test products.

	Formula A Per 100 mL	Formula B Per 100 mL	Cow’s Milk Per 100 mL
Protein (g)	2	2	3.2
Casein (g)	0.97	0.98	2.4
Total Carbohydrates (g)	8.0	8.2	4.6
Lactose (g)	5.63	5.64	4.6
Maltodextrin (g)	2.3	2.5	0
Calcium (mg)	76	81	112
Iron (mg)	1.2	1.2	0.96
Ascorbic Acid (mg)	14	14	1
Prebiotic (scGOS/lcFOS) (g)	0.8	0	0

The chemical composition of formulas was obtained from the label of the products.

Iron isotopes (^59^Fe and ^55^Fe) of high specific activity were used as tracers (Du Pont de Nemours, Wilmington, DE, USA). Milk formulas and ferrous ascorbate aqueous solutions were mixed with isotopes immediately before administration to the subjects. New iron format, where the iron is encapsulated in a carrier material of maltodextrin, stabilized by citric acid was labeled intrinsically during the synthesis. This process was performed by the manufacturer. In brief, the iron was dissolved in food grade distilled water as anhydrous ferrous sulfate (Paul Lohmann, Germany) and ^55^FeCl_3_ or ^59^FeCl_3_ was added as tracer. Then, the iron solution was mixed with citric acid at 70 °C. After 10 min maltodextrin was added at 72% w/w at 45 °C. The specific activity of the labeled iron compound was 555 kBq of ^55^Fe and 185 kBq of ^59^Fe per mg elemental iron.

Milk formulas and ferrous ascorbate were consumed after an overnight fast and no food or beverages other than water were allowed for the following 4 h after the ingestion of the test products. No additional dietary restrictions were provided. On day 1, the subjects ingested 200 mL of milk formula the Formula A, diluted to 14.8%, labeled with 111 kBq of ^55^Fe; on day 2, they ingested 200 mL of the milk formula Formula B, diluted to 14% labeled with 37 kBq of ^59^Fe. A venous blood sample was obtained two weeks later (day 14) to measure the circulating radioactivity and to determine the iron status of the subjects. This same sample also provided baseline values of ^55^Fe and ^59^Fe radioactivity in red blood cells for the next set of absorption studies. On day 14, subjects were given 30 mL of a solution with 3 mg iron and 18.9 mg ascorbic acid labeled with 37 kBq of ^59^Fe, as a reference dose. On day 15, subjects ingested 200 mL of cow’s milk, diluted to 12%, fortified with 8 mg iron per 100 g of powder (as ferrous sulfate) labeled with 111 kBq of ^55^Fe. A final venous sample was obtained on day 28 to determine the increase in red blood cell radioactivity.

**Blood analysis:** Venous blood samples were obtained on day 14 to determine iron status of the volunteers, and on day 14 and 28 to measure circulating radioactivity. Hemoglobin (Hb) and mean corpuscular volume (MCV) were measured by electronic cell counter (CELL-DYN 1700; ABBOTT Diagnostics, Abbott Park, IL, USA). Zinc protoporphyrin (Zpp) was determined in a ZP Hematofluorometer Model 206D; (AVIV Biomedical Inc., Lakewood, NJ, USA) and serum ferritin (SF) was measured by ELISA assay [13].

For the calculation of total radioactivity ingested, aliquots of the compounds were counted in sextuplicates as standards. Measurement of blood radioactivity was performed in duplicate venous samples according to the Eakins and Brown technique [14]. The samples were counted allowing sufficient time period to obtain a counting error of ~3% in a liquid-scintillation counter (Beckman LS 5000 TD; Beckman Instruments, Fullerton, CA, USA). Radioactivity from labeled solution aliquots and venous samples were counted simultaneously at the end of the study to avoid an error in the calculation of iron absorption due to the decay of isotopes between administration and the absorption measurement 14 days later. In addition, absorptions of labeled iron administered on days 14 and 15 were corrected for the isotope given on days 1 and 2 by subtracting the radioactivity of the blood sample of day 14 from red blood cell radioactivity of day 28. The percentages of iron absorption were calculated on the basis of blood volume estimated for height and weight [15], and assuming 80% incorporation of the radioisotope into erythrocytes [16]. This method is reproducible in our laboratory with a coefficient variation of 5%.

**Statistical Analysis:** A sample size of nine subjects was calculated using the software PRIMER, version 3.02, option “power and simple size of analysis of variance (ANOVA)”. The sample size was calculated with an α error of 0.05, a power of 80%, an expected residual standard deviation of three, a number of treatment groups of four and a minimum detectable iron bioavailability difference of 5%. For the study, 15 volunteers were considered in order to account for possible participants lost due to the rejection of intake, and/or the presence of diarrhea or vomiting, producing significant losses of the administered compounds. Because the percentages of iron absorption and serum ferritin had a skewed distribution, these values were log-transformed before calculating means and SDs or performing statistical analyses. Results were re-transformed to recover original units and are expressed as geometric means ±1 standard deviation (SD). The iron bioavailability of each milk formula was corrected to 40% of the iron bioavailability of ferrous ascorbate [17]. Such assessment is made for the purpose of standardizing the comparison of results with other absorption studies. The relationship between the iron absorption and SF was evaluated by a Pearson correlation. Differences in iron absorption were evaluated by a one way ANOVA for repeated measures and *post hoc* Sheffé (Statistica for Windows, release 4.5; StatSoft Inc., Tulsa, OK, USA). All comparisons were done at the 5% level of significance.

## 3. Results

Table 2 shows that the iron nutrition status of the subjects was deficient. A women suffered iron deficiency anemia (Hb < 120 g/L and ≥2 altered biochemical parameters: MCV < 80 fL and/or Zpp > 70 ug/dL RBC and/or %Sat > 15 and/or FS ≤ 12 µg/dL) and other 8 women had iron deficiency without anemia (Hb = normal and ≥ 2 altered biochemical parameters).

**Table 2:** Iron nutrition status of study women.

Subj	Hb	MCV	Zpp	Fe	TIBC	%Sat	SF
	g/L	fL	μg/dLRBC	µg/dL	µg/dL	%	μg/L
1	148	85	73	38	292	13.1	30
2	145	84	69	70	308	22.6	37
3	149	87	74	46	360	12.8	24
4	172	91	46	65	327	19.8	25
5	151	87	74	41	318	12.8	20
6	158	87	66	61	434	14.0	32
7	158	100	63	19	356	5.2	17
8	141	81	106	28	450	6.3	6
9	138	81	114	52	353	14.7	12
10	137	87	86	40	311	12.8	27
11	157	88	60	55	334	16.5	44
12	114	82	134	11	424	2.5	5
13	144	84	83	58	444	13.1	6
14	131	82	54	43	395	10.9	7
15	147	85	83	51	295	17.3	32
Mean	146	86	79	45	360	13.0	17 ^a^
SD	13	5	24	17	56	5.3	8–37

Hb = hemoglobin; MCV = mean corpuscular volume; Zpp = zinc protoporphyrin; Fe = serum iron; TIBC = total iron binding capacity; %Sat = transferrin saturation; SF = serum ferritin; ^a^ Geometric mean and range ±1 SD.

**Figure 1:**
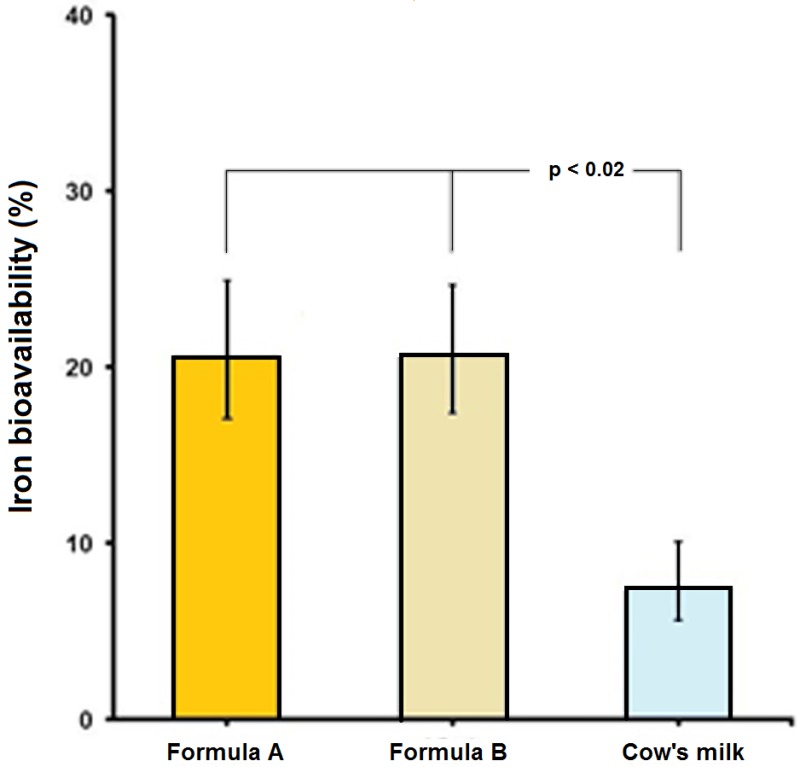
Iron bioavailability of Formula A, Formula B and cow’s milk. The column shows the geometric means corrected to 40% of reference dose and bar shows the ±1 SEM.

Table 3 shows the study’s individual iron bioavailability results. It is observed that the average bioavailability of the reference dose was 38.3% and that it corresponds to the iron absorption of a population deficient in iron, which is similar to the target age group of Formulas A and B. The relationship between the iron bioavailability of the reference dose and the serum ferritin levels of the subjects had a Pearson’s correlation coefficient of 0.79 (*p* < 0.001). The milk formulas A and B had an iron bioavailability of 19.7 and 19.8%, respectively, versus a 7.2% of fortified cow’s milk (one way ANOVA for repeated measures F = 157; *p* < 0.001). The *post hoc* Sheffé test did not show significant differences between Formulas A and B but there were indeed differences between the milk formulas and the cow’s milk (*p* < 0.001). The iron bioavailability of modified milks and cow’s milk had differences with respect to the reference dose of ferrous ascorbate (*p* < 0.001). Upon correcting the iron bioavailability figures of milk products to 40% of the reference dose [17] it is observed that the geometric mean bioavailability for Formulas A and B was 20.6 and 20.7% respectively, versus 7.5% of iron fortified cow’s milk (Figure 1).

**Table 3:** Iron bioavailability of Formula A, Formula B and cow’s milk.

	Iron Bioavailability (%)			
	Formula A	Formula B	Cow milk	Reference dose
S	^55^Fe	^59^Fe	^55^Fe	^59^Fe
1	20.7	29.7	7.3	30.3
2	7.9	8.8	3.7	30.4
3	10.0	10.2	9.5	33.7
4	13.6	5.5	5.3	20.2
5	23.7	25.1	6.1	22.8
6	52.4	55.7	21.8	55.1
7	46.7	37.8	5.1	38.7
8	19.9	13.0	0.3	69.8
9	5.6	9.8	7.2	29.7
10	14.2	17.2	9.5	18.8
11	24.1	23.3	7.9	15.3
12	32.7	33.0	13.5	88.2
13	37.1	32.5	17.1	69.1
14	71.1	58.5	20.2	97.8
15	6.1	11.7	10.4	51.1
GM	19.7	19.8	7.2	38.3
±1 SD	9.0–43.1	9.7–40.5	2.6–19.9	21.4–68.4

GM = Geometric mean; ±1 SD range of +/− 1 standard deviation.

## 4. Discussion

Young children are particularly vulnerable to iron deficiency due to an increase of iron requirements determined by their fast growth [18], inadequate intake of the mineral and/or consumption of low-bioavailability iron. Many young children do not consume large quantities of food rich in bioavailable iron such as red meat. Even in a theoretical model of diet the conclusion was that it is very difficult to reach the recommended intakes of iron with a diet that perfectly adjusts to the food guides for infants and young children [19].

Iron deficiency anemia in children is linked to an increase of morbidity, a reduction in the cognitive development and, therefore, a drop in school activity. It has been evidenced that, when iron deficiency takes place during an early age, the damage to the psychomotor development may be irreversible, even after supplementation with iron [20,21].

Several actions have been proposed as strategies to lower iron deficiency in young children, including the diversification of diets by including food rich in highly-absorbable iron, treatment with anti-parasite medication, and the supplementation and fortification of food with iron [22]. This last strategy has been the most effective one to significantly reduce the prevalence of iron deficiency anemia in children [22,23,24].

The stabilized iron sulfate has been tested in different recipes for infant formulas, follow on formulas, and growing up milks and has proved to be stable for about 1.5–2 years shelf life, preserving the sensory characteristics and stability of fat, including long chain polyunsaturated fatty acids.

This study showed that the iron contained in the milk formulas A and B is very well absorbed, with an average 20% bioavailability. A figure similar to the one previously shown by Hertrampf *et al.*, in bioavailability studies of highly modified commercial infant milk formulas [25]. Regarding the absorption of iron fortified cow’s milk, it must be highlighted that it was similar to what was informed in the literature [11,26]. The only major difference between the Formulas A and B was the first had prebiotics. As iron absorption from both formulas was similar it is concluded that prebiotics did not have an effect on iron absorption. This is consistent with other human studies that demonstrate no effect of these compounds [27,28].

Bioavailability data make it possible to estimate that a toddler consuming 500 mL of Formulas A and B on a daily basis would absorb approximately 1.2 mg of iron per day, which would cover over a 100% of their Recommended Daily Intakes, and which translates into an excellent contribution of this nutrient to the diet of young children [29]. Even if Formulas A and B provide sufficient quantities of iron to young children, there is a need to complement their diet by including meat, fish, fruits, and vegetables so as to provide them with other nutrients such as proteins, unsaturated fats, and vitamins that favor their complete growth and development.

## 5. Conclusions

In conclusion, both A and B milk formulas contain highly bioavailable iron, which contributes to covering the requirements of this micronutrient in toddlers.
